# Reliability, validity and internal consistency of social support and self-efficacy scales for physical activity in adolescents with 10 to 14 years of age

**DOI:** 10.1590/1984-0462/2022/40/2020274

**Published:** 2021-09-01

**Authors:** Gerfeson Mendonça, Alcides Prazeres, Inácio Crochemore-Silva, José Cazuza de Farias

**Affiliations:** a Universidade Federal de Alagoas e Centro Universitário Cesmac, Maceió, AL, Brasil.; b Universidade Federal da Paraíba, João Pessoa, PB, Brasil.; c Universidade Federal de Pelotas, Pelotas, RS, Brasil.

**Keywords:** Motor activity, Adolescent, Social support, Self-efficacy, Validation study, Atividade motora, Adolescente, Suporte social, Autoeficácia, Estudos de validação

## Abstract

**Objective:**

To analyze the reliability, validity and internal consistency of the physical activity social support (SSS) and self-efficacy (SES) scales among adolescents aged 10–14 years.

**Methods:**

The reliability study included 171 adolescents (mean age=12.3±1.0 years, 59.6% female) and validity and consistency study with 1,107 (mean age=12.0±1.0 years, 52.7% female). The SSS had 15 items and the SES eight items. Reliability was determined by Spearman's correlation analysis (rho) and validity and internal consistency by factor analysis (exploratory — EFA and confirmatory — CFA) and the composite reliability (CR), respectively.

**Results:**

The reliability of SSS (father: rho=0.80; p<0.001 | mother: rho=0.76; p<0.001 | friends: rho=0.75; p<0.001) and SES (rho=0.72; p<0.001) were considered high. In the EFA, three factors were identified for the SSS (father, mother and friends — five items each) and one factor for SES (five items), confirmed in the CFA. Quality parameters of adjustment in the final models were satisfactory for SSS (chi-square=240.5 [p<0.001]; root mean square residual — RMR=0.05; root mean square error of approximation — RMSEA=0.04 [90%CI 0.04–0.05]; goodness of fit index — GFI=0.97; adjusted goodness of fit index — AGFI=0.96 and comparative fit index — CFI=0.97) and SES (chi-square=5.4 [p=0.07]; RMR=0.01; RMSEA=0.04 [90%CI 0.00–0.08]; GFI=0.99; AGFI=0.99 and CFI=0.97). Internal consistency was considered satisfactory for SSS (CR: father=0.79, mother=0.77, friends=0.78) and low for SES (CR=0.38).

**Conclusions:**

The scales showed satisfactory levels of reliability and of construct validity. However, only the SSS showed adequate levels of internal consistency.%

## RESUMO

**Objetivo:**

Analisar a reprodutibilidade, a validade e a consistência interna da escala de apoio social (EAS) e da escala de autoeficácia (EAE) para a atividade física em adolescentes de 10 a 14 anos de idade.

**Métodos:**

O estudo de reprodutibilidade contou com 171 adolescentes (12,3±1,0 ano; 59,6% feminino), e o de validade e consistência, com 1.107 (12,0±1,0 ano; 52,7% feminino). A EAS continha 15 itens, e a EAE, oito. A reprodutibilidade foi estimada pela correlação de Spearman (rho), e a validade e a consistência interna, pela análise fatorial (exploratória — AFE e confirmatória — AFC) e pelo índice de fidedignidade combinada (IFC), respectivamente.

**Resultados:**

A reprodutibilidade da EAS (pai: rho=0,80; p<0,001, mãe: rho=0,76; p<0,001 e amigos: rho=0,75; p<0,001) e da EAE (rho=0,72; p<0,001) apresentou forte magnitude. Na AFE, foram identificados três fatores para EAS (pai, mãe e amigos — cinco itens cada) e um fator para EAE (cinco itens), confirmados na AFC (com quatro itens na EAE). Os modelos finais apresentaram indicadores de qualidade satisfatórios para EAS — qui-quadrado=240,5 (p<0,001); *root mean square residual* (RMR)=0,05; *root mean square error of approximation* (RMSEA)=0,04 (intervalo de confiança de 90% — IC90% 0,04–0,05); *goodness of fit index* (GFI)=0,97; *adjusted goodness of fit index* (AGFI)=0,96 e *comparative fit index* (CFI)=0,97 — e EAE — qui-quadrado=5,4 (p=0,07); RMR=0,01; RMSEA=0,04 (IC90% 0,00–0,08); GFI=0,99; AGFI=0,99 e CFI=0,97. A consistência interna foi satisfatória para EAS (índice de fidedignidade combinada — IFC: pai=0,79; mãe=0,77; amigos=0,78) e baixa para EAE (IFC=0,38).

**Conclusões:**

As escalas alcançaram níveis satisfatórios de reprodutibilidade e validade de construto. No entanto, apenas a EAS apresentou níveis de consistência interna adequados.%

## INTRODUCTION

Physical activity (PA) has been associated with benefits related to the physical, mental and social health of adolescents.^1^ However, eight out of ten adolescents in the world do not practice PA according to what is recommended by the World Health Organization (WHO).^2^ ^,^ ^3^ The identification of factors that can influence PA has been considered a priority to enable the implementation of effective interventions to increase PA in adolescents.^2^ ^–^ ^4^ Among these factors are the social support from parents and friends^5^ and the perception of self-efficacy.^2^ ^,^ ^3^

Social support for the practice of PA is characterized by the assistance offered by different social groups (for example, parents, relatives, friends, teachers) to assist in the adoption of this behavior.^6^ Self-efficacy for the practice of PA is defined as the ability of a person to perform some activity even in unfavorable situations (“presence of barriers”), for example, lack of places and/or equipment, motivation and company.^2^ ^,^ ^3^ These constructs are present in theories (socio-cognitive) and models (socioecological) used to explain PA.^2^ ^–^ ^4^ However, they do not provide guidance on how to measure these constructs.^2^

The instruments to assess social support and self-efficacy for PA in adolescents^5^ ^,^ ^7^ ^–^ ^10^ have been tested mostly in young people aged 14 years and over^5^ ^,^ ^7^ from developed countries,^5^ ^,^ ^9^ ^,^ ^10^ who measured only a few items of social support and self-efficacy^8^ ^,^ ^10^ and which did not undergo an assessment of validity levels.^5^ Farias Junior et al.^7^ ^,^ ^11^ developed and analyzed the psychometric aspects of a social support scale (SSS)^7^ ^,^ ^11^ and a self-efficacy scale (SES)^7^ for PA in adolescents aged 14 to 19 years in Northeast Brazil. SSS^7^ ^,^ ^11^ and SES^7^ each showed two factors and reached satisfactory levels of reproducibility, internal consistency and construct validity. These scales were adapted and tested on adolescents aged 11 to 16 years, from the city of Fortaleza, Ceará, remaining with two factors each and with satisfactory levels of reproducibility, internal consistency and construct validity.^12^ However, it is still unknown whether the psychometric quality of these scales is maintained in adolescents aged 10 to 14 years of age, when the assessment of the social support from the father and mother is carried out separately and when the SES uses dichotomous responses for its items, instead of a Likert scale of four points (strongly disagree, disagree, agree, strongly agree).

Pre-teens and younger teenagers (10 to 14 years old) are often involved in a greater variety of PAs compared to older ones, which can imply different barriers to PA practice.^13^ ^,^ ^14^ This impacts the composition of the SES items, considering that they are based on the adolescents’ ability to overcome barriers to the practice of PA. In this sense, it is expected that the sources and types of social support that influence these practices are different from those observed in older adolescents.^15^ Furthermore, studies have identified differences in some types of social support associated with the practice of PA when provided by the father (joint participation and purchase of materials for practice) and by the mother (positive comments and specific incentives).^16^ ^,^ ^17^

In general, younger adolescents have less acute subjective perceptions compared to older ones. It is possible that the use of an SES with dichotomous answer options (yes and no) implies a better understanding of the adolescents about their items, in comparison with the Likert-type scale (strongly disagree, disagree, agree, strongly agree), which can require a higher level of understanding and judgment. Therefore, the aim of this study was to analyze the reproducibility, validity and internal consistency of SSS and SES for PA in adolescents aged 10 to 14 years old.

## METHOD

Two studies were conducted, one to assess reproducibility and the other the construct validity and internal consistency of the SSS and SES for the practice of PA. These scales are part of the questionnaire used to collect data from the Longitudinal Study on Sedentary Behavior, Physical Activity, Food and Adolescent Health - LONCAAFS (Ethics Committee on Human Research at the Federal University of Paraíba (UFPB) - Protocol: 024/13, CAAE: 15268213.0.0000.5188). In both studies, the target population consisted of adolescents from public schools (municipal and state) in the municipality of João Pessoa, Paraíba, Northeast region of Brazil.

To analyze the test-retest reproducibility, a study was carried out with adolescents from the 6th and 8th years of elementary school II — population of 16,746 students (6th year: n=9,520 and 8th year: n=7,226), in 2011. In determination of the sample size, an intraclass correlation coefficient (ICC)≥ 0.20 (reproducibility), type I error of 5%, type II error of 20% (power of 80%) and an increase of 30% for losses and refusals, resulting in a sample of 95 adolescents.

The sample was selected by clusters in two stages:

1. Systematic selection of eight schools — distributed proportionally by type of school (municipal or state) and among the regions of the municipality (north, south, east and west).
2. Random selection of 15 classes (all adolescents of the class were invited to participate in the study).

Data collection was carried out from August to October 2013 by trained staff. The questionnaire was applied in the form of a face-to-face interview, with an interval of two days between applications. The average application time was 25 minutes per adolescent.

The SSS was an adaptation of the Social Support for Physical Activity Scale (ASAFA Scale),^11^ which was composed of ten items: five directed to the social support from parents and five to the support from friends. In this adaptation, five items were added to measure the father's support and five the mother's support, separately. Thus, this version of the scale now had 15 items, five for each source: father, mother and friends. The adolescents reported on the frequency (never, rarely, often, always) with which they received different types of support of PA (for example: they stimulated, practiced along, watched, commented on, and invited [friends] and took [father and mother] to practice) during a typical week.^11^ As an example of an item on the scale, we can quote: “During a normal week, how often does your [father/mother/friends] … [practice] PA with you?”.

The original version of the SES was composed of ten items, on a four-point Likert scale (strongly disagree, disagree, agree, strongly agree).^7^ The items considered how much the adolescents perceived to be able to practice PA under the presence of barriers, for example: “item b — I can practice PA most days of the week, even if I have to pay a fee, monthly fee, bus fare or buy some sporting equipment”. In a pilot study, the adolescents reported difficulties in understanding the Likert scale and suggested that it would be easier to understand and answer if the answer options were “yes” or “no”.

For the characterization of the sample, the variables used were: sex, age (10–12 and 13–14 years), mother's education (incomplete elementary school, complete elementary and high school) and economic class, according to the criteria of the Brazilian Market Research Association (ABEP):^18^ A/B (high) and C/D/E (medium-low).

The frequency distribution was used to describe the sociodemographic variables. The Spearman correlation coefficient (rho) was used to estimate test-retest reproducibility, being classified as: weak (<0.40), moderate (0.40–0.49) and strong (≥0.50).^19^ The reproducibility of SES items was assessed by the agreement coefficient and Kappa Pabak (KPABAK) in: weak (<0.39), moderate (0.40–0.59), excellent (0.60–0.79) and perfect (0.80–1.00).^20^ The analyses were performed on Stata 13.1 (5% significance level).

The construct validity and the internal consistency of the scales were estimated using LONCAAFS baseline data (year 2014), which considered the following parameters for the sample size calculation: 50% prevalence; 95% confidence interval (95%CI); maximum acceptable error of four percentage points; drawing effect of two; and a 40% increase in losses and refusals, resulting in a sample of 1,582 adolescents.

The sample was selected by a single stage cluster: systematic selection of 14 municipal and 14 state schools, distributed proportionally by the region of the municipality and the number of students enrolled in the 6th year. Data collection was carried out from February to December 2014, by the same team, following the same collection protocol of the reproducibility study.

To estimate the construct validity, exploratory factor analyses (EFAs) and confirmatory factor analyses (CFAs) were used.^21^ The EFA was used to determine the number of factors to be extracted and the factor loads of the scale items.^21^ The rotation used was the orthogonal varimax. The criterion suggested by Kaiser^21^ was used to establish the total of factors: values ≥1.0 determined the number of factors in each scale. In SSS, the analysis was performed on the basis of the polychoric correlation matrix (ordinal scale data), and in SES, the tetrachoric correlation (data on nominal scale) was used. The adequacy of the variable items for the analysis of the factors was assessed using the Kaiser Meyer-Olkin index (KMO) and the Bartlett's sphericity test (BST). KMO values were considered satisfactory if they were >0.60 and BST had a level of statistical significance of p<0.05.^21^ Items with factor load ≥0.30 were considered relevant and maintained in the scales.^22^

The CFA was used to assess the quality of the scale model, with its respective factors and items, identified in the EFA. The parameter estimation method was maximum likelihood, based on bootstrap distributions (data without multivariate normal distribution).^23^ The AMOS 20.0 program (analysis of moment structures) was used to perform this analysis.

The quality of fit of the models was assessed using the chi-square test (p>0.05) and the root mean square residual — RMR (≤0.05), root mean square error of approximation — RMSEA (≤0, 05), goodness of fit index — GFI (≥0.90), adjusted goodness of fit index — AGFI (≥0.90) and comparative fit index — CFI (≥0.90).^23^ The values in parentheses indicate levels of acceptable quality. Residual values standardized around ±2.58 and reductions in the values of the chi-square and the RMR, RMSEA, GFI, AGFI and CFI indices were considered as indicators that characterized improvement in the model's quality of fit, in case any parameter was free to be estimated.^23^ ^,^ ^24^

Internal consistency was determined using the combined reliability index (CRI). CRI values ≥0.70 were considered satisfactory.^25^

## RESULTS

The reproducibility study was carried out with 171 adolescents (refusals, losses and exclusions totaled 31.6% of the sample) and the validity and internal consistency study with 1,107 adolescents (refusals, losses and exclusions totaled 24.9% of the sample) of 10 to 14 years old. In the two studies, no significant differences were identified for the sociodemographic variables between those who were included and those excluded from the analszes, except for the variable economic class in the validation and internal consistency study (lower-middle class: excluded=74.0% versus included = 63.5%; p=0.001). Most of the adolescents were female, 10 to 12 years old, of lower-middle income class and children of mothers who did not finish high school (Table 1).

**Table 1:** Sociodemographic characteristics of adolescents in the study of reproducibility (2013) and validity and internal consistency (2014). João Pessoa, Paraíba.

		Study of reproducibility (n=171)		Study of validity and internal consistency (n=1,107)	
		n	%	n	%
Sex					
	Male	69	40.4	524	47.3
	Female	102	59.6	583	52.7
Age group (years)					
	10 to 12	124	72.5	912	82.4
	13 to 14	47	27.5	195	17.6
Economic classᵃ					
	A/B (high)	61	37.0	357	36.5
	C/D/E (medium-low)	104	63.0	621	63.5
Mother's schooling^b^					
	Incomplete elementary school	53	32.7	374	40.0
	Complete elementary school	36	22.2	271	29.0
	Complete high school or more	73	45.1	290	31.0

Items with no answers according to study: reproducibility (ᵃ=6; ^b^=9); and validity and internal consistency (ᵃ=129; ^b^=172).

The reproducibility of the scores of the subscales of social support (father: rho=0.80, p<0.001; mother: rho=0.76, p<0.001; and friends: rho=0.75, p<0.001) was of strong magnitude (Table 2). SES —rho=0.69; p<0.001, before the exclusion of items “e” (KPABAK=0.21) and “g” (KPABAK=0.33) (data not available in tables) — also showed a coefficient of strong reproducibility (rho=0.72; p<0.001).

**Table 2:** Reproducibility of the social support and self-efficacy scales for the practice of physical activity in adolescents. João Pessoa, Paraíba, 2013.

			%C	KPABAK	rho	p-value
Social support for practice of physical activity						
	Father's support					
		Stimulate			0.65	<0.001
		Practice			0.59	<0.001
		Take			0.56	<0.001
		Watch			0.59	<0.001
		Comment			0.63	<0.001
Father's support score					0.80	<0.001
	Mother's support						
		Stimulate			0.56	<0.001
		Practice			0.61	<0.001
		Take			0.50	<0.001
		Watch			0.62	<0.001
		Comment			0.69	<0.001
Mother's support score					0.76	<0.001
	Support from friends						
		Stimulate			0.57	<0.001
		Practice			0.50	<0.001
		Invite			0.56	<0.001
		Watch			0.62	<0.001
		Comment			0.55	<0.001
Support from friends score					0.75	<0.001
	Self-efficacy for practice of physical activity						
		Lack of company	78.4	0.57		
		Must pay some fee	85.4	0.71		
		Other important things	76.5	0.53		
		Do not have locations nearby	80.0	0.60		
		Friends call for other things	60.4	–		
		Do not receive guidance	75.3	0.51		
		Watching TV/playing video games	71.6	–		
		Tired or stressed	83.6	0.67		
Self-efficacy score			–	–	0.72*	<0.001

%C: agreement coefficient; KPABAK: Kappa Pabak; rho: Spearman's correlation coeffecient;

* after excluding items “e” and “g”.

In the EFA, the items of the scales were considered satisfactory for the tolerance of the identity matrix (KMO>0.60) and for the sphericity of the correlation matrix between them (BST <0.05). Three factors for SSS (father, mother and friends) were identified, with five items for each. The factor loads ranged from 0.62 to 0.79 for the father, from 0.67 to 0.77 for the mother and from 0.67 to 0.77 for friends, and the total explained variance was 60.5% (Table 3). SES showed a single factor, and the factor loads ranged from 0.31 to 0.48 for five items (“a”, “b”, “d”, “f” and “h”). The three items with factor load less than 0.30 (“c”=0.23, “e”=0.09 and “g”=0.18 — values not available in the table) were excluded from the scale.

**Table 3:** Exploratory factor analysis of the social support and self-efficacy scales for the practice of physical activity in adolescents. João Pessoa, Paraíba, 2014.

			Exploratory factor analysis			
			Factor 1	Factor 2	Factor 3	Scale
Social support for practice of physical activity						
	Father's support					
		Stimulate	0.69			
		Practice	0.79			
		Take	0.74			
		Watch	0.76			
		Comment	0.62			
	Mother's support					
		Stimulate		0.69		
		Practice		0.77		
		Take		0.74		
		Watch		0.67		
		Comment		0.76		
	Support from friends					
		Stimulate			0.71	
		Practice			0.81	
		Invite			0.74	
		Watch			0.78	
		Comment			0.76	
Number of items			5	5	5	15
% variance explained by the factor			19.7	20.4	20.4	
% total explained variance						60.5
Kaiser Meyer-Olkin (KMO) index						0.79
Bartlett's sphericity test (BST)						<0.001
	Self-efficacy for practice of physical activity†					
		Lack of company	0.48			
		Must pay some fee	0.31			
		Other important things	–			
		Do not have locations nearby	0.45			
		Friends call for other things	–			
		Do not receive guidance	0.41			
		Watching TV/playing video games	–			
		Tired or stressed	0.43			
Number of items						5
Kaiser Meyer-Olkin (KMO) index						0.64
Bartlett's sphericity test (BST)						<0.001

‡ Polychoric correlation matrix;

† tetrachoric correlation matrix.

**Figure 1:**
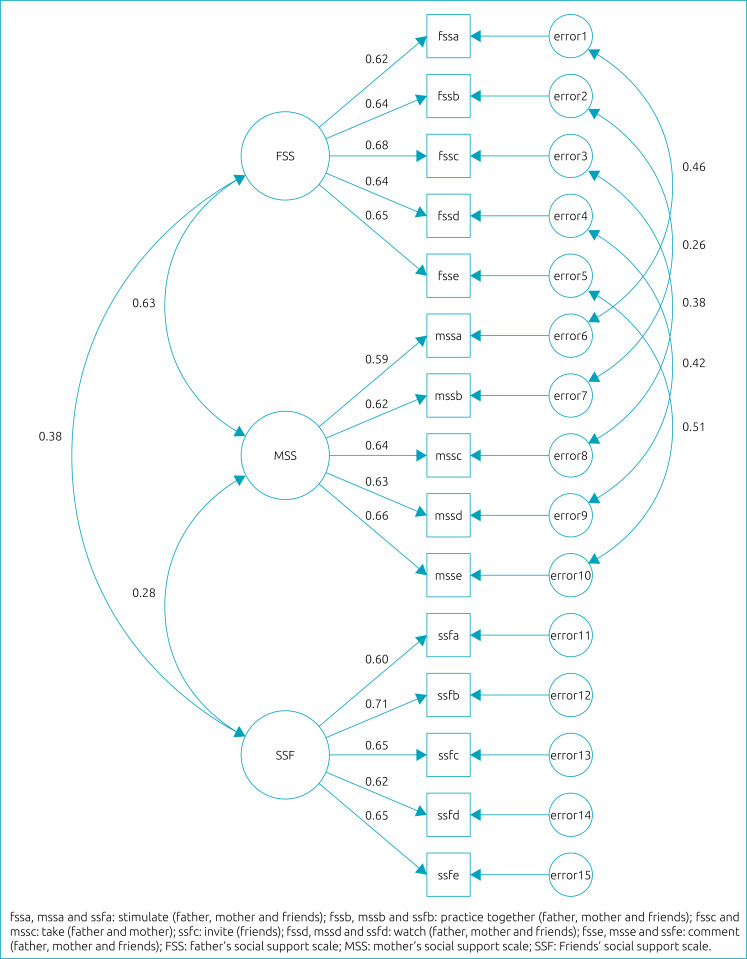
Confirmatory factor analysis of social support scale for father, mother and friends for the practice of physical activity of adolescents. João Pessoa, Paraíba, 2014.

The CFA results for SSS supported the presence of the three factors identified in EFA. All items had factor loads greater than 0.59 (Figure 1). After re-specifying the scale based on the residual values and the covariance between the measurement errors of the items (covariance between the measurement errors of items “a”, “b”, “c”, “d” and “e”), the quality of fit indices were better in all parameters (chi-square=240.5 (p<0.001); RMR=0.05; RMSEA=0.04 (90% confidence interval — 90%CI 0.04 –0.05); GFI=0.97; AGFI=0.96; and CFI=0.97).

**Figure 2:**
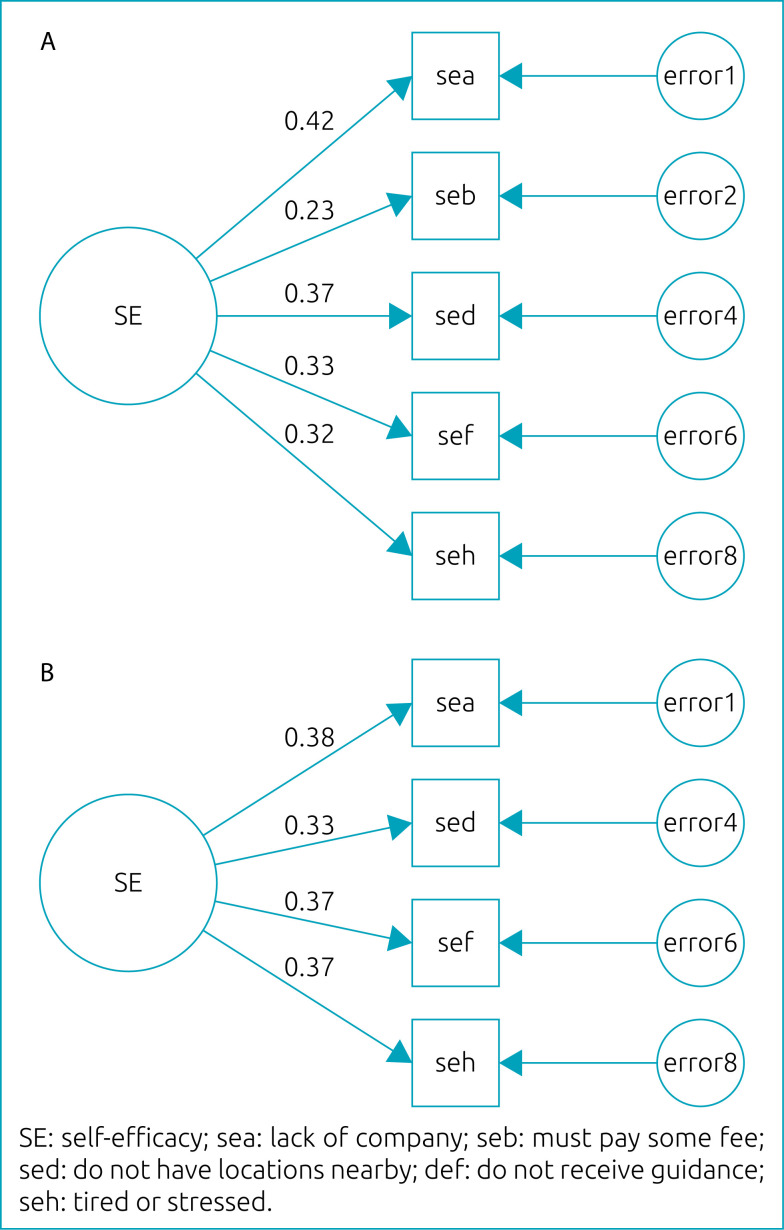
Confirmatory factor analysis of the perception of self-efficacy scale for the practice of physical activity in adolescents. João Pessoa, Paraíba, 2014.

In SES, AFC corroborated the presence of a single factor, with five items and factor loads ranging from 0.23 to 0.42 (Figure 2A). After re-specifying the model (exclusion of item “b” due to the high value of the residue and the low factor load), the factor loads ranged from 0.33 to 0.38 (Figure 2B), and the adjustment index values were the following: chi-square=5.4 (p=0.07); RMR=0.01; RMSEA=0.04 (90%CI 0.00–0.08); GFI=0.99; AGFI=0.99; and CFI=0.97.

The internal consistency of the social support subscales was: CRI=0.79 for the father; CRI=0.77 for the mother; and CRI=0.78 for friends, indicating satisfactory levels. SES, on the other hand, showed unsatisfactory internal consistency (CRI=0.38) — information not presented in tables or figures.

## DISCUSSION

The SSS and SES for the PA practice analyzed reached satisfactory levels of reproducibility and construct validity. However, only SSS showed adequate levels of internal consistency.

The reproducibility levels of SSS (father, mother and friends) and SES (general) reached values above the recommended minimum (rho≥0.50),^20^ similar to those found in other studies.^27^ ^,^ ^12^ In SES, low levels were identified reproducibility levels for items “e” (being able to practice PA even if your friends call you to do other things except PA) and “g” (being able to practice PA even if you could stay at home watching TV, playing video game or using the computer). This may be due to the use of two or more activities as conditions for the practice of PA, which may have compromised the clarity and objectivity of the questions in these items. The exclusion of these items improved the reproducibility level of the scale score, going from rho=0.69 (before exclusion) to rho=0.72 (after exclusion).

The results of EFA and CFA (construct validity) indicated the presence of three factors for SSS (father, mother and friends), with five items for each factor, all with factor loads greater than 0.59. This is an important finding, considering that providing social support for the practice of PA in adolescents can be carried out by different sources of support. In a systematic review, Mendonça et al.^5^ identified that there is a wide variety of sources of social support (parents, friends, siblings, uncles, cousins, grandparents, nephews/nieces, teachers and managers). However, the support of the father, mother and friends is characterized as the main sources associated with adolescents’ PA. These are the subgroups that have the greatest contact with adolescents and have an important economic and social influence on different behaviors (including PA)^26^ and can provide different types of social support (instrumental, psychological and instructional)^27^ and vary according to the type of PA to be practiced and sex and age of the adolescent.^5^ This subsidizes and reinforces the importance of measuring social support with specific items for each source of support.

Studies have shown that the social support provided by the father and mother can specifically influence the practice of PA.^5^ ^,^ ^16^ ^,^ ^17^ The father's support has been shown to be more important for male adolescents, and the mother's support for female adolescents, and this relationship can still vary according to the types of support provided by each of these sources.^16^ ^,^ ^17^ Therefore, it is necessary that the scales to assess social support for the practice of PA in adolescents consider in their structure the support provided separately by the father, mother and friends.

It is also important to highlight that correlations were observed between the residuals of the items of the subscales of support of the father and mother. This result can be explained by the similarity of the items (the same items were asked for father and mother) and by the possible interdependence in the provision of these types of support between these sources. However, these sources have a particular influence on the adolescents’ PA, and the quality of the model increased when these items were considered separately (items from the father and items from the mother) to measure social support.

The results of the EFA indicated that the SES was one-dimensional and composed of five of the eight items proposed in the initial version. The exclusion of items “c”, “e” and “g”, due to the low factor loads, reflects their low contribution to the scale measurement, which may be due to the lack of clarity in the formulation of the question. Barbosa Filho et al.^12^ also identified low factor loads and made exclusions for items similar to the ones in the present study, which reinforces the possibility of low comprehension of these by the adolescents. Consideration should also be given to the possibility that the adolescents answered what would be more socially accepted/appropriate in relation to prioritizing activities, such as school tasks, in the case of item “c”, and having greater pleasure or preference for sedentary activities (for example, watching television and playing video games), to the detriment of the practice of PA, as referred to in item “g”.

An important aspect to be highlighted is that, although the dichotomization of the SES items was pointed out in the pilot study as a possible facilitator for the understanding of the adolescents, the factor loads of the remaining items were lower than those of the items presented in other studies.^7^ ^,^ ^12^ This suggests that the barriers used to compose the items on this scale may not have been representative for the adolescents studied. However, after re-specifying the items in the model, the remaining factor loads met the recommended criteria (≥0.30),^22^ and the other psychometric parameters (RMR, RMSEA, GFI, AGFI and CFI) showed levels considered satisfactory.

The number of dimensions identified in the SES of this study was the same as that identified in another scale tested on Chinese adolescents^28^ and different from what was observed in studies that used the same scale of the present study in other Brazilian adolescents, but with a four-point Likert scale (strongly disagree, disagree, agree, strongly agree).^7^ ^,^ ^12^ Ling et al.^28^ identified in AFC the presence of a dimension in SES in Chinese children and adolescents aged 8 to 12 years. Farias Júnior et al.^7^ identified two dimensions for SES in adolescents aged 14 to 19 years old (four items — resources for the practice of PA; and six items — social support and reasons for the practice of PA), and Barbosa Filho et al.^12^ confirmed the presence of two dimensions (five items — individual and social barriers; and three items – resources for the practice of PA) in schoolchildren aged 11 to 16 years.

One possible explanation for the differences in the number of dimensions may be related to the fact that the items of the instruments to measure self-efficacy are based on barriers for a specific practice of PA.^2^ ^,^ ^3^ In this sense, it is possible that the barriers used to compose the items of the scale may not be representative of the adolescents who were part of the present study, considering the age group and the socioeconomic profile (predominantly lower-middle class people). Studies have indicated that the barriers to the practice of PA change with age.^26^ The PA most practiced by these adolescents may not require places, materials, specific equipment and/or money. Thus, they can perceive other barriers that were not considered in the scale. It must be considered that the dose (quantity) of PA practice conditioned to the ability to overcome barriers to its performance may not have been clear to adolescents. They may also have overestimated the amount of practice and considered that it would be easier to adopt other behaviors that are more pleasurable or easier to adopt, such as watching TV, using the computer and playing video games.

In SSS, the levels of internal consistency were considered satisfactory for the three sources (father, mother and friends), but were below that recommended for SES. Similar results were found in studies that used the same SSS in adolescents aged 14 to 19 years old^11^ and from 13 to 14 years old.^12^ A high level of internal consistency indicates that the items used represent well the measured construct — social support for the practice AF. The low levels of internal consistency of SES reinforce what has already been mentioned about the possible lack of representativeness of the items (barriers to the practice of PA) in this population group (young people and lower economic class). It is necessary to develop new studies with this scale, using the focus group strategy to identify barriers of different dimensions (environmental, social, psychological, cognitive and emotional) that are representative for a certain dose or level of PA practice in younger adolescents.

One of the limitations of the present study was that it did not include students from the private school system. It has been observed that the perception of self-efficacy and barriers to the practice of PA is different according to the socioeconomic conditions of adolescents.^29^ In general, adolescents from the private network are from families with better socioeconomic conditions^26^ and have higher levels of PA at leisure.^14^ Within this domain of practice, they usually carry out structured activities,^14^ which may be more affected by the presence of barriers. Another limitation was that the SES questions were not previously tested for clarity and objectivity. In the present study, most adolescents (about 80%) were 10 to 12 years old. It is possible that their PA practice and their ability to understand the issues are different from their peers aged 14 to 19 years, for which these scales were developed.

Two important consequences can be mentioned when using a scale with low levels of internal consistency:^30^

1. The items on the scale may not correctly express the construct to be measured.
2. The measurement error estimate can be higher, considering that the variability of the results can affect the power of the statistical tests, increasing the probability of non-significant results.

SSS and SES for PA practice achieved satisfactory levels of reproducibility and construct validity. However, internal consistency was high only for SSS. These scales can be used to measure psychosocial factors (self-efficacy and social support) for the practice of PA in adolescents aged 10 to 14 years old. However, it is recommended to exercise caution when interpreting the data obtained by SES, as it did not reach satisfactory levels of internal consistency.

## References

[1] Patton GC, Sawyer SM, Santelli JS, Ross DA, Afifi R, Allen NB (2016). Our future: a Lancet commission on adolescent health and wellbeing. Lancet.

[2] Bauman AE, Reis RS, Sallis JF, Wells JC, Loos RJ, Martin BW (2012). Correlates of physical activity: why are some people physically active and others not?. Lancet.

[3] Sallis JF, Bull F, Guthold R, Heath GW, Inoue S, Kelly P (2016). Progress in physical activity over the Olympic quadrennium. Lancet.

[4] Li YC, Joshi D, King-Dowling S, Hay J, Faught BE, Cairney J (2018). The longitudinal relationship between generalized self-efficacy and physical activity in school-aged children. Eur J Sport Sci.

[5] Mendonça G, Cheng LA, Melo EN, Farias JC (2014). Physical activity and social support in adolescents: a systematic review. Health Educ Res.

[6] Glanz K, Rimer BK, Viswanath K (2008). Health behavior and health education: theory, research, and practice.

[7] Farias JC, Lopes AS, Reis RS, Nascimento JV, Borgatto AF, Hallal PC (2011). Development and validation of a questionnaire measuring factors associated with physical activity in adolescents. Rev Bras Saúde Matern Infant.

[8] Lubans DR, Morgan PJ (2009). Social, psychological and behavioural correlates of pedometer step counts in a sample of Australian adolescents. J Sci Med Sport.

[9] Ren Z, Hu L, Yu JJ, Yu Q, Chen S, Ma Y (2020). The influence of social support on physical activity in Chinese adolescents: the mediating role of exercise self-efficacy. Children.

[10] Bachner J, Sturm DJ, Haug S, Demetriou Y (2020). Multi-level validation of the German physical activity self-efficacy scale in a sample of female sixth-graders. BMC Public Health.

[11] Farias JC, Mendonça G, Florindo AA, Barros MV (2014). Reliability and validity of a physical activity social support assessment scale in adolescents – ASAFA Scale. Rev Bras Epidemiol.

[12] Barbosa VC, Rech CR, Mota J, Farias JC, Lopes AS (2016). Validity and reliability of scales on intrapersonal, interpersonal and environmental factors associated with physical activity in Brazilian secondary students. Rev Bras Cineantropom Desempenho Hum.

[13] Hoefer WR, McKenzie TL, Sallis JF, Marshall SJ, Conway TL (2001). Parental provision of transportation for adolescent physical activity. Am J Prev Med.

[14] Mendonça G, Cheng LA, Farias JC (2018). Standards of physical activity practices in adolescents in a city of North-eastern Brazil. Ciênc Saúde Coletiva.

[15] Mendonça G, Farias JC (2015). Physical activity and social support in adolescents: analysis of different types and sources of social support. J Sports Sci.

[16] Peterson MS, Lawman HG, Wilson DK, Fairchild A, Horn ML (2013). The association of self-efficacy and parent social support on physical activity in male and female adolescents. Health Psychol.

[17] Wenthe PJ, Janz KF, Levy SM (2009). Gender similarities and differences in factors associated with adolescent moderate-vigorous physical activity. Pediatr Exerc Sci.

[18] Associação Brasileira de Empresas de Pesquisa (2012). Brazil Economic Classification Criterion.

[19] Hulley SB, Cummings SR, Browner WS, Grady DG, Newman TB (2015). Outlining clinical research.

[20] Landis R, Koch GG (1977). The measurement of observer agreement for categorical data. Biometrics.

[21] Hair JF, Black WC, Babin BJ, Anderson RE, Tatham RL (2009). Multivariate data analysis: a global perspective.

[22] Matsunaga M (2010). How to factor-analyze your data right: do's, don'ts, and how-to's. Int J Psychol Res.

[23] Kline R (2005). Principles and practice of structural equation modelling.

[24] Byrne BM (2010). Structural equation modelling with AMOS: basic concepts, applications, and programming.

[25] Helms JE, Henze KT, Sass TL, College B, Mifsud VA (2006). Treating Cronbach's alpha reliability coefficients as data in counselling research. Couns Psychol.

[26] Seabra AF, Mendonça DM, Thomis MA, Anjos LA, Maia JA (2008). Biological and socio-cultural determinants of physical activity in adolescents. Cad Saude Publica.

[27] Beets MW, Vogel R, Forlaw L, Pitetti KH, Cardinal BJ (2006). Social support and youth physical activity: the role of provider and type. Am J Health Behav.

[28] Liang Y, Lau PW, Huang WY, Maddison R, Baranowski T (2014). Validity and reliability of questionnaires measuring physical activity self-efficacy, enjoyment, social support among Hong Kong Chinese children. Prev Med Reports.

[29] Cocker K, Artero EG, De Henauw S, Dietrich S, Gottrand F, Beghin L (2012). Can differences in physical activity by socio-economic status in European adolescents be explained by differences in psychosocial correlates?. A mediation analysis within the HELENA (Healthy Lifestyle in Europe by Nutrition in Adolescence) study. Public Health Nutr.

[30] Maroco J, Garcia-Marques T (2006). How reliable is Cronbach's alpha? Old issues and modern solutions?. Lab Psciol.

